# When insect development meets insect succession: Advantages of combining different methods and insect taxa in estimating the post-mortem interval

**DOI:** 10.1016/j.fsisyn.2025.100574

**Published:** 2025-01-21

**Authors:** Szymon Matuszewski, Anna Mądra-Bielewicz

**Affiliations:** a Laboratory of Criminalistics, Adam Mickiewicz University, Al. Niepodległości 53, 61-714, Poznań, Poland; b Center for Advanced Technologies, Adam Mickiewicz University, Uniwersytetu Poznańskiego 10, 61-614, Poznań, Poland

**Keywords:** Forensic entomology, Post-mortem interval, Pre-appearance interval, Insect age, Estimation errors

## Abstract

Insects are frequently used to estimate post-mortem interval (PMI). Experts usually base their estimates on a single insect taxon and use a single estimation method, even if multiple taxa are present on a cadaver or multiple methods can be applied. In this article we present a case report where multiple insect evidence and methods were used in a homicide case to estimate PMI. Since the true PMI was known, we were able to assess the estimation errors of each method as applied to particular insect evidence. The final grand PMI was derived from a developmental estimate based on third instar larvae of *Lucilia illustris*/*L. caesar* species group and a successional estimate based on adult *Thanatophilus rugosus* beetles. By averaging these estimates we got the grand PMI of almost perfect accuracy (1 % relative error, PMI range: 4.39 ± 0.77 days), which is of course an exceptional situation for entomological methods of estimating PMI. Furthermore, this was the first case report in which the presence and absence of subsequent life stages of carrion insects coupled with the estimation of their pre-appearance interval were used to estimate the PMI range. The results regarding the minimum PMI were fully consistent with the results obtained using the classical developmental method. This finding indicates that in some cases the presence/absence method can be used interchangeably with the developmental method. Finally, we discussed the prospects and limitations of combining insect evidence and methods of their analysis in estimating PMI.

## 1 Introduction

Insects present at the death scene are very useful in determining the circumstances of death, especially the post-mortem interval (PMI). In general, PMI can be estimated using the development or succession of insects [1,2]. Various methods were proposed for this purpose. Regarding insect development, PMI is traditionally estimated based on the developmental advancement of immature insects sampled from the cadaver. This advancement can be measured by the grand developmental landmarks reached by the insects [3,4], size of larvae [[5], [6], [7]], morphological landmarks of pupae [8,9] or by the further rearing of larvae or pupae in the laboratory [10,11]. As for insect succession, PMI is estimated from the successional advancement of the cadaver entomofauna. It can be analysed using the presence of two definitive taxa, as evaluated using insect occurrence data [[12], [13], [14]], the presence and absence of subsequent life stages of carrion insects coupled with the estimation of their pre-appearance interval (PAI) [15], the presence/absence of insect taxa, as modelled with respect to the degree-day accumulation [16] or the presence of the most successionally advanced insect taxon coupled with the estimation of its PAI [17].

Apart from different methods, there is a variety of insect taxa that can be used for the estimation of PMI. Blow flies (Calliphoridae) are the most commonly used for this purpose worldwide, followed by flesh flies (Sarcophagidae), muscid flies (Muscidae), skipper flies (Piophilidae) and scuttle flies (Phoridae) among Diptera, as well as skin beetles (Dermestidae) and rove beetles (Staphylinidae including former Silphidae) among Coleoptera, with several number of used species in most of these families [[18], [19], [20], [21], [22]]. Hu and colleagues [18] found that among 307 examined case reports, on average three species were mentioned: 2.7 when PMI was estimated based on the development and 5.1 when succession was used for this purpose.

Despite this variety, single methods and taxa are usually used in the practice of forensic entomology. Most frequently, experts estimate PMI using insect development data of larvae and pupae or puparia [18]. The simultaneous application of development and succession patterns is hardly performed [23,24]. Furthermore, it is infrequent for multiple insect taxa use, and even if, experts usually choose only one for the final conclusion or combine estimates derived using different taxa to extend the PMI range [[23], [24], [25], [26]]. Therefore, a typical scenario in casework is to base the PMI on a single insect taxon and use a single estimation method, even if multiple taxa are available or multiple methods can be applied. To some extent, this state is justified. The monopolization of a cadaver by one species is not an uncommon phenomenon [19,27]. Moreover, due to the non-professional collection of insect evidence, experts frequently receive entomological material that is not very diverse. However, based on the results of study by Hu et al. [18], it can still be concluded that they usually have access to insect evidence of several species.

Therefore, an urgent question is, how should we proceed when multiple insect evidence or multiple methods can be used to estimate PMI? A recent validation study showed that averaging estimates across multiple pieces of insect evidence yielded a systematically more accurate PMI than using a single piece of evidence [28]. Combining different insect-based methods is probably similarly beneficial. It is known that supplementing insect age with PAI makes the resultant estimated PMI generally closer to the true PMI [23,24,28]. However, this is the case when a single, albeit compound, method is used. Regarding the use of different insect-based methods, each yielding its own PMI estimate, we still have no data on the potential benefits of combining such estimates. In a different research field, Henssge and colleagues [29] found that applying a temperature-based nomogram method in combination with various non-temperature-based methods for short PMI considerably improved the accuracy of estimated PMI range.

Although in most cases the decision to use multiple pieces of insect evidence or methods is likely to be appropriate, there is no guidance on which evidence or methods to combine and how to do it, what benefits to expect, and what limitations to consider. With the current case report, we illustrate possibilities, benefits and limitations when different methods and insect species are used to estimate PMI in a homicide case.

## 2 Case report

### 2.1 Case circumstances

The body of an adult female homicide victim was discovered on June 19^th^ inside a mixed forest near Poznań (Western Poland). The body was naked and laying on its back on the forest floor. Death occurred in another place and the body was transferred to the forest shortly after death. It was generally in a fresh or initial bloated decay stages, in the face there were signs of active decay *sensu* Payne [30]. The pathologist estimated PMI to be longer than 72 hours.

### 2.2 Cadaver entomofauna

Fly larvae were collected by law enforcement officers from nasal openings during the body inspection at the place of its discovery. We also received the reports (along with photographic documentation) of the inspection of the place where the body was found and the inspection of the body in the place where it was found, as well as the autopsy report.

**Fig. 1:**
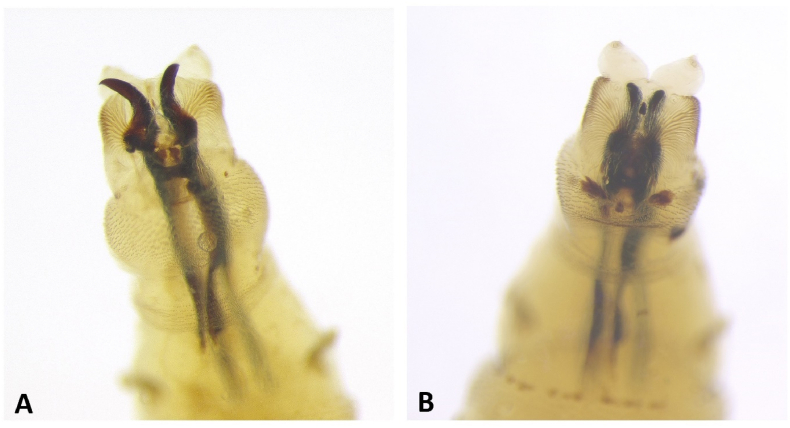
Pseudocephalon of the feeding third instar larvae of *Lucilia caesar*/*Lucilia illustris* – A, and *Lucilia ampullacea* – B. Specimens were collected from the nasal openings of the body during its inspection in the place where it was found.

**Fig. 2:**
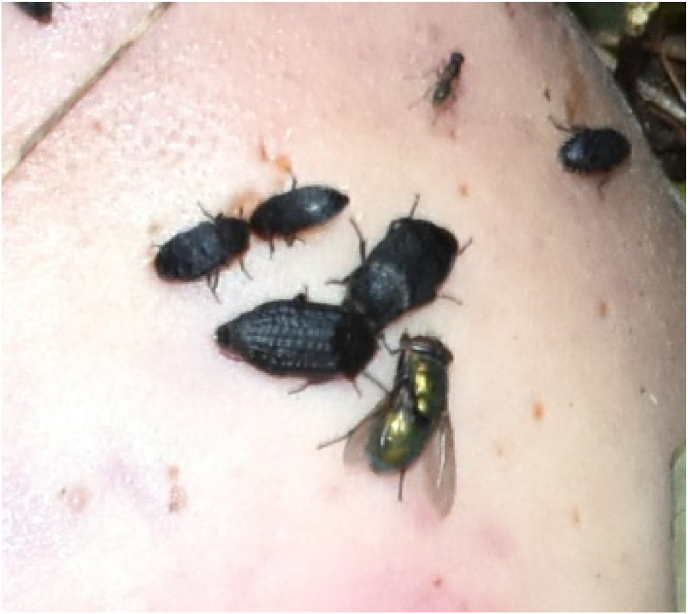
Adult beetles of *Thanatophilus rugosus* and *Dermestes murinus* and some adult flies present on the cadaver (picture taken during the inspection of the cadaver in the place of its discovery).

We identified 40 feeding third instar larvae of *Lucilia ampullacea* Villeneuve, 1922 and 5 feeding third instar larvae of the *L. caesar* Linnaeus, 1758/*L. illustris* (Meigen, 1826) species group (Fig. 1; Table 1). There were also few first and second instar larvae of blow flies (Calliphoridae). Moreover, in the pictures taken during the inspection of the body in the place where it was found, we identified adult beetles of *Thanatophilus rugosus* (Linnaeus, 1758), *Dermestes murinus* Linnaeus, 1758 and *D.* *undulatus* Brahm, 1790, several taxa of adult flies and numerous eggs and larvae of flies (Fig. 2; Table 1).

**Table 1:** Insect taxa collected or recorded on the cadaver.

Order	Family	Genus/species	Life stage	N	Origin
Diptera	Calliphoridae	*Lucilia ampullacea*	L3	40	S
		*L. caesar*/*L. illustris*	L3	5	S
		–	L1	3	S
		–	L2	2	S
		*Calliphora* sp.	A	–	P
		*Lucilia* sp.	A	–	P
	Muscidae	*Hydrotaea* sp.	A	1	P
	Piophilidae	–	A	1	P
	–	–	E, L1-L3	–	P
Coleoptera	Staphylinidae	*Thanatophilus rugosus*	A	–	P
	Dermestidae	*Dermestes murinus*	A	–	P
		*Dermestes undulatus*	A	–	P

A-adult insects, E-eggs, L1-first instar larvae, L2-second instar larvae, L3-third instar larvae.

S-sample, P-picture, N-number of recorded specimens.

### 2.3 Temperature conditions

We obtained meteorological data from the local station (Poznań-Ławica) recorded between June 1^st^ and June 19^th^ (Table 2). To account for the systematic thermal differences between the station and the place where the cadaver was found, we retrospectively corrected temperature recordings from the station. For this purpose temperature was recorded in the place where the body was found: from September 18^th^ until September 23^rd^ using two HOBO U23 Pro v2 2x External Temperature Data Loggers (Onset Computer Corporation, MA, USA). Tree cover, the most common reason of systematic thermal differences between forests and weather stations, was very similar during the recording period and on the day of the cadaver discovery. Therefore, the three-month period from 19^th^ June probably had only a negligible effect on the accuracy of temperature reconstruction in this case. Our records were compared using linear regression with records from the station and the model equation was used for the correction.

**Table 2:** Temperature data from the local meteorological station (before and after correction) and thermal accumulation data used while estimating the age of blow fly larvae.

Date	Temperatures at the station [°C]				*Lucilia caesar*		*Lucilia illustris*	
	Before correction			After correction				
	Max	Min	Mean	Mean	DD > 8.3 °C	ADD>8.3 °C	DD > 9.68 °C	ADD>9.68 °C
June 10	21.5	11.4	16.6	15.44	7.14	70.615	5.76	57.505
11	19.1	13.1	14.1	12.93	4.63	63.475	3.25	51.745
12	18.9	8.7	13.8	12.63	4.33	58.845	2.95	48.495
13	16.8	7.9	12.2	11.02	2.72	54.515	1.34	45.545
14	20.1	6.1	14.8	13.63	5.33	51.795	3.95	44.205
15	24.6	14	18.6	17.45	9.15	46.465	7.77	40.255
16	24.8	13.6	19.5	18.36	10.06	37.315	8.68	32.485
17	25	15.2	19.7	18.56	10.26	27.255	8.88	23.805
18	28	15.1	22.5	21.37	13.07	16.995	11.69	14.925
June 19	26.4	14.8	17.3	16.15	7.85/2 = 3.925	3.925	6.47/2 = 3.235	3.235

Max – maximum recorded temperature, Min – minimum recorded temperature, DD – degree-days, ADD – accumulated degree-days.

**Fig. 3:**
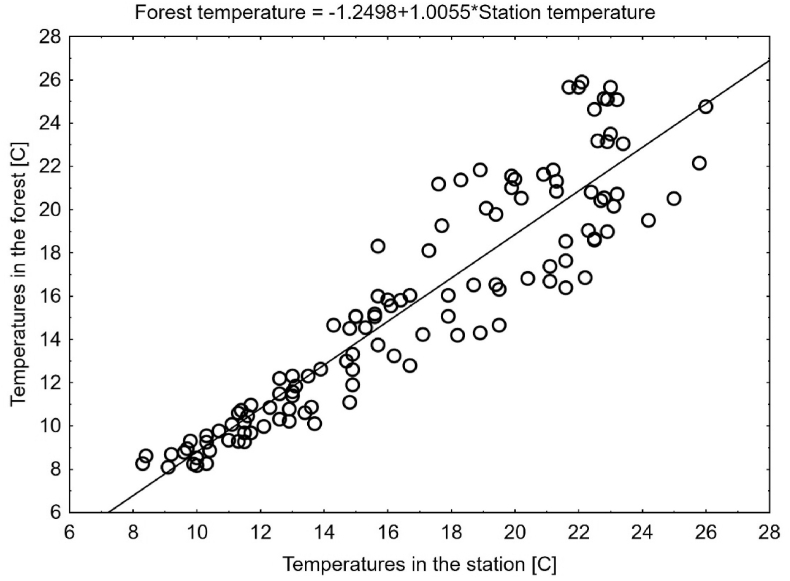
Correlation between temperature records in the station and in the forest where the body was found.

There was a strong correlation between temperatures in the forest and at the station (linear regression, Forest temperatures = −1.2498 + 1.0055∗Station temperatures, *r*^*2*^ = 0.84, Fig. 3). Temperatures in the place where the cadaver was found were systematically lower by about 1.2 °C (Table 2).

### 2.4 PMI estimation

#### 2.4.1 Insect development

**Fig. 4:**
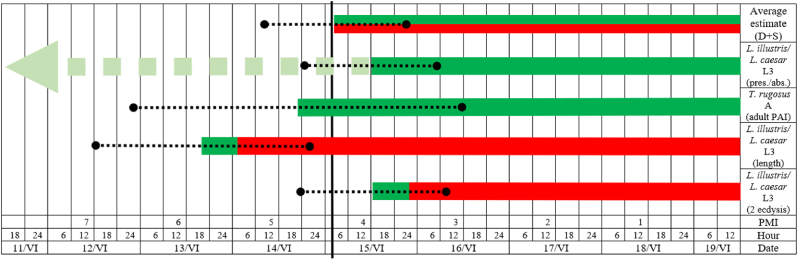
Accuracy of particular estimation methods. Red bars – developmental estimates (D), green bars – successional estimates (S), dotted lines – PMI ranges, vertical bold line – the true PMI, L3 – third instar larvae, A – adult insects.

First, we used the thermal summation method to estimate the time necessary to reach the second ecdysis by larvae of *L. caesar*/*L. illustris* species group. Since there are no developmental data for *L. ampullacea* these larvae were not used in the estimations. As for the *L. caesar*/*L. illustris* larvae we applied two data sets, one for *L. caesar* [31] and the other for *L. illustris* [32]. Using the first data set, we assumed a developmental threshold for the second ecdysis of 8.3 °C and a thermal constant for this landmark of 35.1 ADD. The estimated chronological age of the larvae was 3.28 days (Table 2). For the *L.* *illustris* data set, the developmental threshold was 9.68 °C, the thermal constant was 35.5 ADD (we added constants for the egg, L1 and L2 stages) and the estimated chronological age was 3.89 days (Table 2). Since we used two data sets, estimated ages were averaged, which yielded the final estimate of 3.58 days. To incorporate the oviposition PAI (an interval preceding oviposition of the species), we used PAI data for *L. caesar* [33]. June 15^th^ and 16^th^ were warm days, mean temperatures (after correction) were 18 °C (minimum temperatures at the station for these days were 13–14 °C and maximum about 24–25 °C). Under such conditions and in the circumstances of this case, blow flies should oviposit without a delay, resulting in short oviposition PAI. Following the published PAI patterns for *L. caesar*, we assumed 0.4 day for blow fly oviposition PAI. Therefore, the most probable estimated PMI based on *L. caesar*/*L. illustris* larvae was 3.98 days. To transform it into PMI range, we used average error rate of 20 % [17,34]. The estimated PMI range was 3.18–4.78 days (3.98 ± 0.8 days, Fig. 4).

Second, we estimated the age of *L. caesar*/*L. illustris* larvae using measurements of larval length and isomegalen diagram developed for *L. illustris* by Wang and colleagues [32]. There is no such diagram for *L. caesar*. The examined material contained larvae in the 11–13 mm length range. The average temperature for six days preceding the discovery of the body was 17.6 °C. Using the diagram, the age of 13 mm *L. illustris* larvae was estimated at about 130 hours, which is 5.42 days. Assuming 0.4 day for the oviposition PAI, the most probable estimated PMI based on the length of *L. caesar*/*L. illustris* larvae was 5.82 days. Using 20 % average error rate, the PMI range estimated in this way was 4.66–6.98 days (5.82 ± 1.16 days, Fig. 4). In this case, errors are probably much larger due to the way the larvae were preserved (45 % ethanol solution without the recommended prior killing in hot water) and processed in the laboratory (10 % KOH solution to improve the visibility of diagnostic features) [[35], [36], [37], [38]]. Therefore, we decided not to use this estimate in the further analyses and final conclusions.

#### 2.4.2 Insect succession

The third estimate was for adult *T. rugosus* and its PAI approximated by the temperature method. In June the average PAI for these beetles is 6.7 days (Matuszewski, unpublished data). The average temperature for this 7-day period preceding June 19^th^ was 16.65 °C. Using this temperature as a predictor variable and a temperature model for the PAI of adult *T. rugosus* [39], its PAI was estimated to be 6.1 days. After two iterations, the estimated PAI stabilized at 4.8 days. Since the average error of PAI estimation for adult beetles is 37 % [17], based on the PAI of adult *T. rugosus* we got the PMI range of 3.02–6.58 days (4.8 ± 1.78 days, Fig. 4).

Finally, we applied for the first time in casework the successional approach developed by Matuszewski [15], in which the presence and absence of subsequent life stages delineates the PMI range. In this case, the taxon present was a feeding form of the third instar larvae of the *L. caesar*/*L. illustris* species group*.* Its PAI was estimated with the model for the feeding third instar larvae of *L. caesar* (Table 2 in ESM2 of [15]). In June its average PAI is 7.3 days (Matuszewski, unpublished data). The average temperature for the 7-day period preceding June 19^th^ was 16.65 °C. With this temperature as a predictor variable and using the above model, we obtained a PAI of 5.4 days in the first estimate. After two iterations, we received a PAI of 4 days, which is the estimated minimum PMI. The absent taxon was a postfeeding form of the third instar larvae of *L*. *illustris*/*L. caesar*. Using the model for the PAI of the postfeeding third instar larvae of *L. caesar* (Table 2 in ESM2 of [15]), its average June PAI of 9.8 days (Matuszewski, unpublished data) and the average temperature for the 10-day period of 15.75 °C, we obtained a PAI of 10.2 days in the first estimate. Because iterations could not change this result, 10.2 days was the estimated maximum PMI. Therefore, the estimated PMI range was 4–10.2 days (Fig. 4). While the maximum PMI is of little use in this case (the larvae were quite far from reaching the postfeeding migratory form), the minimum PMI is fully consistent with the results of the developmental estimation. To define the uncertainty of this estimate, we used the average error rate of 18 % appropriate for larval PAI [17] and obtained the range of 3.28–4.72 days (Fig. 4).

#### 2.4.3 Combining developmental and successional estimates

The developmental and the second variant of successional (presence/absence) estimation were based on the same insect evidence, i.e. a feeding form of the third instar larvae of *L. illustris*/*L. caesar* species group. Therefore, it is not surprising that these estimates are consistent regarding the most probable PMI and the PMI range (Fig. 4). The first variant of successional estimation was based on adult *T. rugosus* beetles. Both the estimation method and the insect evidence used were different in this case. A recent validation study of entomological methods for estimating PMI demonstrated that the estimation accuracy improved when estimates were averaged for some pieces of insect evidence [28]. Averaging estimates across early life stages of late colonizing species and late life stages of early colonizing species or across just early life stages of late colonizing species was particularly beneficial. The average error for such grand PMI estimates (including adult PAI) was about 13 % [28]. Therefore, we decided to average a developmental estimate based on the third instar larvae of *L. illustris*/*L. caesar* species group with a successional estimate based on adult *T. rugosus*. In this way, we obtained 4.39 days as the most probable estimated PMI. The above-mentioned validation study showed that grand PMI estimates have an average error of 12.8–22.8 % (17.6 % on average) depending on the type of averaged estimates [28]. When we used the average error rate of 17.6 %, the obtained PMI range was 3.62–5.16 days (4.39 ± 0.77 days, Fig. 4).

## 3 Discussion

The true PMI was determined based on the suspect's statement. It was 4.42 days (4 days and 10 hours). The authors were aware of the suspect's statement during the analyses. Because this knowledge could have influenced the course of the analyses, we made every effort to ensure that no such influence occurred. We believe that our analyses and reasoning (detailed, step by step in this report) confirm that the knowledge of the true PMI did not influence our judgments. The true PMI allowed us to calculate the true errors of each method and each piece of insect evidence applied in this case and thus to highlight the strengths and weaknesses of particular insect-based PMI estimates (Table 3).

**Table 3:** Accuracy of particular insect-based estimates.

	Insect evidence		Applied method	PMI estimate [days]		Covering of the true PMI	Error	
	Species	Life stage		Point	Range		Absolute [days]	Relative [%]
1	*L. illustris*/*L. caesar*	L3	D (2^nd^ ecdysis) + PAI	3.98	3.18–4.78	YES	−0.44	11
2			D (length) + PAI	5.82	4.66–6.98	NO	1.4	24
3			S (pres./abs.)	7.1	4–10.2	YES	2.68	38
4	*T. rugosus*	A	S (PAI)	4.8	3.02–6.58	YES	0.38	8
5	1 and 4		D and S	4.39	3.62–5.16	YES	−0.03	1

A-adult insects, L3-third instar larvae, D-developmental method, S-successional method, YES-the estimated PMI range covers the true PMI, NO-the estimated PMI range does not cover the true PMI.

The only method that gave a PMI range which did not cover the true PMI was the developmental estimate based on larval length (Table 3). There were two important sources of error related to its application in this case. First, the larvae were improperly preserved (without the hot water killing and with only 45 % ethanol solution used for their preservation), which must have affected the accuracy of length measurements. Previous studies have generally found that larvae shrink, when they are directly transferred to the preservative without first killing them in hot water [35,38,40]. On the other hand, a recent study has shown that some larvae can increase in length when stored in ethanol [37]. Thus, when fly larvae are improperly fixed or preserved their measurements will inaccurately represent their true length and inevitably lead to smaller or larger errors in the estimation of their age. Moreover, there is no method to incorporate these errors and calibrate age estimates accordingly. Second, in the analyses we could only use the isomegalen diagram developed for *L. illustris* from China [32]. Because geographic populations of the same species may differ in terms of growth rate and insect size [[41], [42], [43], [44]], the current use of developmental data for a distant population may have increased the error in age estimates. Regardless of the reasons for the errors in the current estimates, this case shows that estimating age of larvae based on their length requires caution.

In this case, for the first time, the presence and absence of subsequent life stages of carrion insects were used to estimate the PMI range according to the method proposed by Matuszewski [15]. The resultant minimum PMI was fully consistent with the developmental estimates, indicating that in some cases this method can be used interchangeably with the classical developmental method (Fig. 4). The maximum PMI was long, resulting in a wide PMI range and the largest error of all methods applied in this case (Table 3). Larvae were far from reaching the postfeeding stage; furthermore, temperatures between 11^th^ and 14^th^ June were low, which increased the estimated PAI for this stage. This resulted in a long maximum PMI, which was of little use in this case. Nevertheless, the presence/absence method has proven useful and should be further developed by identifying new configurations of indicators and collecting related PAI data.

Not surprisingly, the classical developmental method (i.e. estimating the time necessary to reach a developmental landmark, here supplemented with the oviposition PAI) was accurate, giving a narrow PMI range and a small error (Table 3). Grand developmental landmarks (i.e. hatching, ecdyses, pupariation/pupation and eclosion) are generally easily determined based on the morphology or size measurements [[45], [46], [47]]. Moreover, there are robust datasets regarding the time needed to reach these landmarks for many forensically useful insects (e.g. Refs. [[48], [49], [50]]). Therefore, when insects are collected shortly after reaching a landmark, this method usually allows their age to be accurately estimated, as this case clearly demonstrates. Due to these reasons, this method is probably the most commonly used entomological approach to estimating PMI and the present case confirms its high usefulness for the practice of forensic entomology.

We were surprised by the good results of the PAI method when it was applied to estimate PMI based on the presence of adult *T. rugosus* beetles. Although it gave a wider PMI range than the classical developmental method, its error was smaller (Table 3). Adult insects are rarely used to estimate PMI, this case demonstrates that they can be as useful as immature insects under certain circumstances. *Thanatophilus* beetles can abundantly colonize animal or human cadavers [[51], [52], [53]]. In the present case, only a few of these insects were documented during the cadaver inspection. Therefore, the residency of *T. rugosus* must have started shortly before the body disclosure, and thus their PAI could provide an accurate approximation of the PMI. These beetles can be present on cadavers for a long time and, as with other carrion insects of forensic importance, their residency increases with an increase in cadaver mass [[54], [55], [56]]. Therefore, their presence on a cadaver, especially in a more advanced state of decomposition will generally be less useful for estimating PMI than it was in this case. Nevertheless, the present case demonstrates that adult carrion insects can be used to estimate PMI, when their residency on a cadaver has only just begun and when good PAI data are available.

The most fascinating aspect of this case report was the combination of estimates for multiple insect evidence and using different methods and its effect on the accuracy of a resultant grand PMI. We combined a classical developmental estimate based on the feeding third instar larvae of *L. illustris*/*L. caesar* species group with a successional estimate based on adult *T. rugosus.* The resultant grand PMI had the lowest error and the narrowest PMI range. Of course, one should not expect that near-perfect accuracy of the present estimate (1 %) will be a rule. In fact, such accuracy is an exception when using insect evidence for estimating PMI. Usually, lower accuracy of insect-based methods is the case, with average errors of about 10–20 % [18,34]. However, this example shows that entomological methods for estimating PMI can be very accurate, even despite certain weaknesses of the reference data or errors in collecting or preserving insects. Although there is no data on the benefits of averaging developmental and successional estimates derived based on different insect evidence, averaging compound estimates (i.e. insect age supplemented with PAI) for different insect evidence was greatly beneficial in terms of the accuracy of the grand PMI, with the average error of 13 % [28]. The current case report clearly adds to these findings by emphasizing that priority should be given to averaging PMI estimates derived using different methods and based on different pieces of insect evidence.

Several questions arise regarding the use of multiple methods and insect evidence. First, which estimates (for which insect evidence) can be combined in the way that was done here. We believe that it is beneficial to combine PMI estimates derived for insect evidence that co-occur during succession. Combining evidence from insects colonizing cadavers early in succession with evidence from middle or late colonizers is probably the most beneficial (Table 4). However, combinations of evidence from different species in the same colonizer category (e.g. full puparia of different blow fly species or larvae of different late colonizers) can also be useful. A thorough understanding of insect succession on cadavers in a given habitat and geographical area will be necessary to wisely average PMI estimates based on different insect evidence. Second, what benefits can be expected following the use of multiple evidence or methods. Averaging PMI estimates will be most beneficial when overestimations are combined with underestimations, because their errors will cancel each other out after calculating the grand PMI. In fact, this is what happened in the present case. On the other hand, combining different overestimations or underestimations will only produce a more robust PMI estimate (due to its embeddedness in multiple evidence), but without reducing the estimation error meaningfully. Third, what risks can be expected from using multiple evidence or methods. By combining PMI estimates that differ substantially in accuracy, we may reduce the quality of the grand PMI. In particular, insect evidence that poorly represents PMI may reduce the gains in accuracy provided by other evidence e.g. when full blow fly puparia are present, using their larvae in combination with third instar larvae of *Necrodes littoralis* (L.) will usually have a detrimental effect on the accuracy of the grand PMI. Therefore, when individual PMI estimates differ substantially from each other or when there is no good justification that the combined pieces of insect evidence co-occur during succession, averaging estimates to obtain a grand PMI requires great caution.

**Table 4:** Examples of insect evidence configurations useful for combining PMI estimates.

Configuration type			Examples of taxa^a^
No	Life stages	Colonizers	
I	Larvae	Early	L3 of *Lucilia caesar, Calliphora vomitoria* etc.
	Adults	Middle/late	Adults of *Thanatophilus* sp.*, Necrodes littoralis, Stearibia nigriceps* etc.
II	Larvae	Early	L3/PF of *L*. *caesar, C*. *vomitoria* etc.
	Larvae	Middle	L1, L2 of *Thanatophilus* sp. etc.
III	Full puparia	Early	*L*. *caesar, C*. *vomitoria* etc.
	Larvae	Middle/late	L1-L3 of *Thanatophilus* sp.*, N*. *littoralis, S*. *nigriceps* etc.
IV	Empty puparia/tenerals	Early	*L*. *caesar, C*. *vomitoria* etc.
	Larvae	Late	L3 of *N*. *littoralis, S*. *nigriceps* etc.
V	Empty puparia/tenerals	Early	*L. caesar, C*. *vomitoria* etc.
	Full puparia	Late	*S. nigriceps* etc.

a – examples based on patterns of insect succession on pig carcasses in Central European forests [14], L1 – first instar larvae, L2 – second instar larvae, L3 – third instar larvae, L3/PF postfeeding third instar larvae.

In summary, this case report demonstrates the advantages of using multiple insect evidence and combining developmental and successional methods to estimate PMI in a homicide case. By averaging the PMI estimated developmentally based on third instar larvae of *L. illustris*/*L. caesar* species group with the PMI derived successionally based on adult *T. rugosus* beetles, we got the grand PMI of almost perfect accuracy. In addition, this is the first case report in which the presence and absence of subsequent life stages of carrion insects were used to estimate PMI, and one of very few cases in which adult insects were used for this purpose. Furthermore, the present case exemplifies the benefits of retrospective correction of temperatures for use in PMI estimation and the opportunities that knowledge of the true PMI provides in assessing estimation errors.

## CRediT authorship contribution statement

**Szymon Matuszewski:** Writing – review & editing, Writing – original draft, Visualization, Methodology, Investigation, Conceptualization. **Anna Mądra-Bielewicz:** Writing – review & editing, Visualization, Methodology, Investigation.

## Declaration of competing interest

The authors declare that they have no known competing financial interests or personal relationships that could have appeared to influence the work reported in this paper.
